# The use of filler DNA for improved transfection and reduced DNA needs in transient gene expression with CHO and HEK cells

**DOI:** 10.1186/1753-6561-5-S8-P33

**Published:** 2011-11-22

**Authors:** Divor Kiseljak, Yashas Rajendra, Sagar S Manoli, Lucia Baldi, David L Hacker, Florian M Wurm

**Affiliations:** 1 Laboratory for Cellular Biotechnology, Faculty of Life Sciences, École Polytechnique Fédéral de Lausanne, CH-1015 Lausanne, Switzerland

## Background

Transient gene expression (TGE) is a rapid method for the production of recombinant proteins. Protein productivity in TGE has improved significantly over the past decade, reaching 300 mg/L and 1 g/L in CHO DG44 (CHO) and HEK 293E (HEK) cells, respectively [1,2]. However, the amount of plasmid DNA needed for transfection remains relatively high, contributing significantly to the overall cost of the TGE process. In order to reduce the amount of plasmid DNA in TGE, we examined the possibility of partially replacing it with herring sperm DNA (non-coding “filler” DNA) in transfections of CHO and HEK cells.

## Materials and methods

### Transfections

Suspension-adapted CHO were centrifuged and resuspended in ProCHO5 (Lonza, Verviers, Belgium) at a density of 4 x 10^6^ cells/mL. Transfections of 5 mL were performed in TubeSpin^®^ 50 bioreactors (TPP, Trasadingen, Switzerland) using 0.625 µg DNA/1x10^6^ cells and 2.5 µg/1x10^6^ cells of linear 25 kDa polyethyleneimine (PEI; Polysciences, Eppenheim, Germany). pA3 carrying the genes for a human IgG light and heavy chains was used for transfections [1]. The transfected cultures were incubated at 31 °C in 5% CO_2_ and 85% humidity with agitation at 180 rpm. HEK cells were centrifuged and resuspended at a density of 20 x 10^6^ cells/mL in RPMI 1640 medium (Lonza). To each culture, 1.0 µg DNA/1x10^6^ cells and 3.0 µg PEI/1x10^6^ cells were added. At 3 h post-transfection, cells were diluted with Ex-Cell293^TM^ medium (Sigma) medium to a density of 1 x 10^6^ cells/mL, and valproic acid was added to a final concentration of 3.75 mM. The transfected cultures were incubated at 37 °C as above.

### Analyses

The IgG concentration was determined by sandwich ELISA [3]. To quantify plasmid DNA copy number, total cellular DNA was extracted using DNeasy Blood & Tissue Kit (Qiagen) according to the manufacturer’s protocol. To estimate the mRNA transgene levels, total RNA was extracted from cells using the GenElute mRNA kit (Sigma) according to the manufacturer’s protocol. DNA-free RNA was reverse transcribed using M-MLV reverse transcriptase (Sigma) and oligo dT as the primer. The RT-qPCR was carried out in a LightCycler 480 Real-Time PCR System (Roche Applied Science, Basel, Switzerland) with the ABsolute QPCR SYBR Green ROX mix (Thermo Fisher Scientific) according to the manufacturer’s instructions.

## Results

### Filler DNA allows considerable reduction in coding pDNA amounts

**Figure 1:**
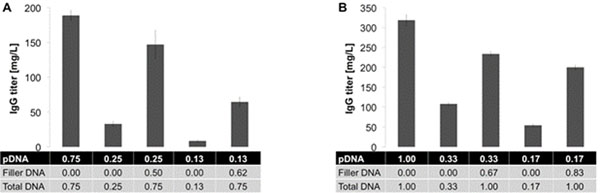
Effect of filler DNA on transient IgG production in A) CHO and B) HEK cells. IgG titers were measured on day 7 post-transfection by ELISA. The DNA amounts are presented in μg/10^6^ cells.

We tested the efficiency of herring sperm DNA as filler for TGE in CHO and HEK cells. We reduced the amount of pA3 to 17% or 33% of the optimum amount for each cell line and added filler DNA to 100%. The total amount of PEI was kept constant for all conditions. We observed that antibody titers increased when filler DNA was co-transfected with pA3 as compared to transfection with a reduced amount of pA3 alone (Fig. 1). These results showed that up to 83 % of the coding pDNA could be replaced by filler DNA with only a minimal negative impact on yield.

### Filler DNA does not influence the delivery of coding pDNA

We investigated whether the use of filler DNA could improve pDNA delivery and/or intracellular pDNA stability by quantifying the plasmid copy number by qRT-PCR. The results showed that the plasmid copy number decreased proportionally with the amount of pA3 transfected in the presence or absence of filler DNA in both CHO and HEK cells (data not shown). Therefore, filler DNA did not influence the delivery of coding pDNA to transfected cells.

### Filler DNA enhances transgene mRNA levels and improves release of coding pDNA

We tested the hypothesis that the use of filler DNA could influence transcriptional competence of coding pDNA. By qPCR, we found that transgene mRNA levels were significantly higher in the presence of filler than in its absence (data not shown). This may explain the improved protein titers observed in the presence of filler DNA. We then hypothesized that filler DNA could influence the strength of PEI:DNA complexes and pDNA release from the complex upon delivery. With an in vitro dextran sulfate displacement assay we observed that with increasing PEI:DNA ratios, complex strength increased. However, when filler DNA was added to the complex, the release of pDNA from the complex was improved (data not shown).

## Conclusions

Our data show that in TGE the amount of the coding vector could be reduced considerably by replacement of a significant proportion of pDNA with filler DNA (herring sperm DNA) without a major negative impact on recombinant protein productivity. However, filler DNA did not influence the delivery or stability of pDNA. The addition of filler DNA to the DNA-PEI complex, however, relaxed the complex *in vitro*. Based on these results, we speculate that the presence of filler DNA results in a more efficient intracellular release of the pDNA from the DNA-PEI complex and thus to improved transgene transcription.
